# Improved hospitalization rates in a specialty center for heart failure with preserved ejection fraction and pulmonary hypertension

**DOI:** 10.1002/pul2.12002

**Published:** 2022-06-07

**Authors:** Chad M. Kosanovich, Hongyang Pi, Adam Handen, Erin Schikowski, Yimin Chen, Floyd W. Thoma, Suresh Mulukutla, Steve Koscumb, Mehdi Nouraie, Stephen Y. Chan

**Affiliations:** ^1^ Department of Medicine, Division of Cardiology University of Pittsburgh Medical Center Pittsburgh Pennsylvania USA; ^2^ Department of Medicine, Division of Pulmonary, Critical Care and Sleep Medicine University of Washington Medical Center Seattle Washington USA; ^3^ Center for Pulmonary Vascular Biology and Medicine; Pittsburgh Heart, Lung, Blood, and Vascular Medicine Institute Pittsburgh Pennsylvania USA; ^4^ Department of Medicine University of Pittsburgh Medical Center Pittsburgh Pennsylvania USA; ^5^ Clinical Analytics University of Pittsburgh Medical Center Pittsburgh Pennsylvania USA; ^6^ Department of Medicine, Division of Pulmonary, Allergy and Critical Care Medicine University of Pittsburgh Pittsburgh Pennsylvania USA

**Keywords:** combined post‐ and precapillary PH, heart failure with preserved ejection fraction, pulmonary hypertension, specialty care center

## Abstract

Heart failure with preserved ejection fraction can be complicated by pulmonary hypertension. We designed a retrospective study to provide supporting evidence for referral to specialty care centers. Specialty care centers improved hospitalizations but not mortality—in part due to more aggressive medication management and guideline‐directed monitoring.

**Abbreviations**
Cpc‐PH: combined post‐ and precapillary PH
HFpEF: heart failure with preserved ejection fraction
HC: hazard ratio
Ipc‐PH: isolated postcapillary PH
mPAP: mean pulmonary arterial pressure
PH: pulmonary hypertension
RHC: right heart catheterization
SCC: specialty care centers
UPMC: University of Pittsburgh Medical Center

## BACKGROUND

Heart failure with preserved ejection fraction (HFpEF) accounts for nearly half of hospitalizations for heart failure in the United States.^1^ Chronic passive venous congestion raises postcapillary pulmonary pressures and can induce pulmonary hypertension (PH) with worsened prognosis.^2^, ^3^ In HFpEF registries, the prevalence of PH‐HFpEF is noted up to 83%.^2^

Passive transmission of venous congestion can cause isolated postcapillary PH (Ipc‐PH).^4^ As pulmonary vascular disease becomes disproportionate to left ventricular disease, a combined post‐ and precapillary PH (Cpc‐PH) phenotype develops.^5^ Cpc‐PH resembles pulmonary arterial hypertension with respect to genetics, hemodynamics, and survival, but comorbidities mimic Ipc‐PH.^4^, ^5^

Unlike pulmonary arterial hypertension and more similar to the management of HFpEF, PH‐HFpEF lacks guideline‐directed vasodilator therapy, and management focuses on the optimization of comorbidities and volume status.^6^, ^7^ In the United States, the Pulmonary Hypertension Association has increased referral efforts to specialty care centers (SCC) with mortality and hospitalization benefits for pulmonary arterial hypertension.^8^, ^9^ Current guidelines recommend referral to SCC when a Cpc‐PH phenotype develops, but guidelines lack supporting data with IIIc level of evidence.^6^

We compared care of PH‐HFpEF managed at SCC versus non‐SCC locations and hypothesized SCC improved outcomes with respect to mortality and hospitalizations with higher compliance using contemporary guidelines for pulmonary hypertension.^6^

## METHODS

The Institutional Review Board at the University of Pittsburgh Medical Center (UPMC) approved this study (PRO11070366), and this study was performed in accordance with the Helsinki Declaration. By using the Medical Archival Retrieval System, a repository of UPMC health data encompassing 41 network hospitals, patients were retrospectively identified between January 1, 2008, and December 1, 2018, with a left ventricular ejection fraction ≥50% by transthoracic echocardiogram with a mean pulmonary arterial pressure (mPAP) ≥25 mmHg and pulmonary capillary wedge pressure >15 mmHg by index right heart catheterization (RHC). Patients were further classified by RHC using pulmonary vascular resistance. Ipc‐PH was defined when pulmonary vascular resistance was <3 woods units. Cpc‐PH was defined when pulmonary vascular resistance was ≥3 woods units. Patients with at least one encounter with a SCC provider at UPMC (accredited by the Pulmonary Hypertension Association) were included in the SCC cohort. The non‐SCC cohort was further divided, based on a hospital size cut‐off of 300 beds.

Demographics, comorbidities by International Classification of Disease codes, yearly medications, and frequencies of RHC and transthoracic echocardiogram were included from index RHC. A mixed effect survival model with the random coefficient for each patient and Weibull distribution established time to hospital admission. Cox proportional hazard modeling was used for mortality and presented as hazard ratio (HR) with adjustment for disease severity using mPAP, age, gender, and comorbidities. Medication adjustment was used in subanalysis with 83% coverage in the cohort as well as hospital size. Two‐sided statistical tests with a *p* < 0.05 was defined as significant. All statistical analyses were performed using Stata 17.0 software (StataCorp).

## RESULTS

A total of 2863 patients in the UPMC system met inclusion criteria with 974 managed at SCC and 1889 at non‐SCC. SCC patients were younger (median 66 vs. 69 years, *p* < 0.001), more commonly female (60 vs. 51%, *p* < 0.001), followed for longer (5.3 vs. 4.1 years, *p* < 0.001), and had worsened hemodynamic parameters including mPAP and pulmonary vascular resistance (*p* < 0.001). Pulmonary capillary wedge pressure was lower for SCC (*p* < 0.001). Cardiac output was not different. The frequencies of hypertension, congestive heart failure, coronary artery disease, obesity, chronic obstructive pulmonary disease, obstructive sleep apnea, pulmonary fibrosis, end‐stage renal disease, cirrhosis, and thromboembolism were significantly higher in SCC. SCC management led to more frequent RHCs (mean: 1.22, 95% CI: 1.16–1.29 vs. 1.06, 95% CI: 1.04–1.07, *p* < 0.001) but not echocardiograms.

Referral and management by SCC were associated with reduced hospital admissions (HR: 0.84, 95% CI: 0.78–0.90, *p* < 0.001) (Table 1), but not mortality. When subdivided for hemodynamic group, Cpc‐PH (HR: 0.84, 95% CI: 0.74–0.94, *p* = 0.003) and Ipc‐PH (HR: 0.83, 95% CI: 0.76–0.92, *p* < 0.001) demonstrated a significant reduction in hospitalizations without mortality difference. After index RHC, 87 patients in SCC and 40 in non‐SCC converted from Ipc‐PH to Cpc‐PH. In two additional analyses, including completely removing the converts and reclassifying all converters into the Cpc‐PH subgroup, these major significant differences in SCC versus non‐SCC outcomes were still observed. A secondary analysis to stratify based on mPAP ≥40 from index RHC demonstrated a more robust reduction in hospitalization for SCC overall (HR: 0.77, 95% CI: 0.66–0.91, *p* = 0.002) and when subdividing for Cpc‐PH (HR: 0.78, 95% CI: 0.64–0.94, *p* = 0.012) and Ipc‐PH (HR: 0.64, 95% CI: 0.50–0.84, *p* = 0.001) specifically.

**Table 1:** Effects of SCC on clinical outcomes including hospitalization and mortality

	Effect on time to admission (HR)^d^	Effect on mortality (HR)^d^	Effect on time to admission (HR)^c^	Effect on mortality (HR)^c^
1a: SCC vs. non‐SCC centers (large and small)				
All^a^				
(*N* = 2602)^d^	**0.84 (0.78–0.90**	1.09 (0.95–1.24	**0.89 (0.82**–**0.97**	1.08 (0.93–1.25
(*N* = 2153)^c^	***p*** < **0.001)**	*p* = 0.21)	***p*** = **0.007)**	*p* = 0.33)
Cpc‐PH				
(*N* = 1009)^d^	**0.84 (0.74**–**0.94**	1.08 (0.89–1.31	**0.87 (0.76**–**0.99**	1.09 (0.88–1.35
(*N* = 870)^c^	***p*** = **0.003)**	*p* = 0.44)	***p*** = **0.030)**	*p* = 0.43)
Ipc‐PH				
(*N* = 1593)^d^	**0.83 (0.76**–**0.92**	1.06 (0.88–1.29	0.91 (0.82–1.01	1.05 (0.84–1.30
(*N* = 1283)^c^	***p*** < **0.001)**	*p* = 0.52)	*p* = 0.08)	*p* = 0.70)
1b: SCC vs. large non‐SCC centers				
All^b^				
(*N* = 2123)^d^	**0.84 (0.77**–**0.91**	1.09 (0.94–1.25	**0.86 (0.79**–**0.94**	1.05 (0.90–1.23
(*N* = 1893)^c^	***p*** < **0.001)**	*p* = 0.25)	***p*** < **0.001)**	*p* = 0.51)
Cpc‐PH				
(*N* = 849)^d^	**0.84 (0.74**–**0.95**	1.15 (0.93–1.42	**0.81 (0.71**–**0.93**	1.05 (0.84–1.31
(*N* = 772)^c^	***p*** = **0.005)**	*p* = 0.19)	***p*** = **0.003)**	*p* = 0.667)
Ipc‐PH				
(*N* = 1274)^d^	**0.84 (0.76**–**0.93**	1.0 (0.82–1.22	**0.89 (0.80**–**0.98**	–
(*N *= 1121)^c^	***p*** < **0.001)**	2.0 *p* = 0.98)	***p*** = **0.024)**	*p* = 0.83)

*Note*: Bold values denote *p* < 0.05.

^a^ Adjusted for age, gender, mean pulmonary arterial pressure, and baseline comorbidities including congestive heart failure, chronic obstructive pulmonary disease, coronary artery disease/coronary artery bypass grafting, obstructive sleep apnea, pulmonary fibrosis, end‐stage renal disease, cirrhosis, deep vein thrombosis/pulmonary embolism.

^b^ Adjustment as above including medications: loop diuretics, spironolactone, vasodilators, antihypertensives, antiplatelets, statins, anticoagulation, diabetes treatment, obstructive airway treatment.

^c^ Interaction between center and group: *p* = 0.56 for time to admission, *p* = 0.55 for mortality when compared to all hospitals (1a).

d Interaction between center and group: *p* = 0.40 for time to admission, *p* = 70 for mortality when compared to large hospitals (1b).

Medication use differed among cohorts, especially with respect to loop diuretics, spironolactone, and vasodilator therapy. SCC patients were more commonly prescribed loop diuretics (47% vs. 40%, *p* = 0.001) and spironolactone (18% vs. 10%, *p* < 0.001), and significant for each hemodynamic group for loop diuretics (Cpc‐PH: 51% vs. 45%, *p* < 0.001 and Ipc‐PH: 48% vs. 42%, *p* < 0.001), and spironolactone (Cpc‐PH: 20% vs. 10%, *p* < 0.001 and Ipc‐PH: 16% vs. 11%, *p* < 0.001). Vasodilator use was more common in SCC (14% vs. 1.5%, *p* < 0.001) and most evidently in Cpc‐PH (21% vs. 2.7%, *p* < 0.001). There was low frequency use in Ipc‐PH (3.8% vs. 0.9%, *p* < 0.001). Of SCC patients, 12 Ipc‐PH patients were on vasodilators, with most vasodilators being phosphodiesterase inhibitors prescribed for erectile dysfunction (four patients) or for Cpc‐PH hemodynamics that appeared later and with documented improvement in symptoms (eight patients). Alternatively, 10 patients in non‐SCC Ipc‐PH subgroup were prescribed vasodilators, with the majority started on vasodilator therapy inappropriately (six patients) and a minority prescribed such medications for erectile dysfunction (four patients).

To address the possibility that SCC versus non‐SCC outcomes were driven by these differences in medication use, analyses were performed of patients with available medication records. Adjustment for medication use resulted in a significant but modestly mitigated reduction in hospital admission (adjusted HR: 0.89, 95% CI: 0.82–0.97, *p* = 0.007) for SCC patients (Table 1a). When subdivided by hemodynamic group, a significant yet mitigated reduction in hospitalizations was still seen for Cpc‐PH (HR: 0.87, 95% CI: 0.76–0.99, *p* = 0.03), but was nonsignificant in Ipc‐PH.

To address the possibility that SCC versus non‐SCC outcomes were driven by differences in access to large hospital facilities, we compared the SCC cohort to only non‐SCC patients managed at other large hospitals. In this context, hospital admissions were again reduced in the SCC (HR: 0.86, 95% CI: 0.79–0.94, *p* < 0.001) (Table 1b). When subdivided by hemodynamic group, this reduction of hospital admission was observed in both Cpc‐PH (HR: 0.81, 95% CI: 0.71–0.93, *p* = 0.003) and Ipc‐PH (HR: 0.89, 95% CI: 0.80–0.98, *p* = 0.024) groups. When adjusting for medications, improvement in admissions was only partially blunted, indicating that medications play a part of improving admissions, but do not explain the total benefit of the SCC.

## DISCUSSION

This retrospective, single‐hospital system analysis elucidates crucial differences in care for PH‐HFpEF patients provided at an SCC and offers evidence of improved hospitalizations. This improvement became more evident in PH patients with mPAP ≥40, suggesting those with severe disease benefit robustly from referral. More aggressive treatment of volume status and comorbidities might improve symptoms and hospitalizations in SCC patients.^6^, ^7^, ^10^ This is reflected in Table 1a, as adjustment for medications resulted in a mitigated improvement in hospitalizations related to SCC care. When comparing hospitalizations between the SCC and other large non‐SCC hospitals in Table 1b, the adjustment for medication use was less apparent. This may be indicative of two important points. First, medical management at SCC significantly improves outcomes. Second, this improvement in hospitalizations is partially driven by medication adjustment and partially related to greater adherence to disease management guidelines by the SCC. Published guidelines for managing both HFpEF and PH‐HFpEF emphasize consistent volume optimization.^6^, ^11^ The pulmonary capillary wedge pressure at SCC was lower than non‐SCC locations suggesting better control of volume status. Particularly in Cpc‐PH patients known to carry worsened prognoses,^4^ such improvements may be related to the SCC's high compliance with guideline‐directed disease monitoring,^6^ as reflected by the higher frequencies of RHC utilization for proper diagnosis and serial hemodynamic follow‐up.^12^ Additionally, spironolactone use has been emphasized in HFpEF,^10^, ^13^ and more frequent use was noted in the SCC (Cpc‐PH: 20% vs. 10%, *p* < 0.001 and Ipc‐PH: 16% vs. 11%, *p* < 0.001). Notably, a small subset of patients with pulmonary vascular disease was prescribed vasodilators, which may provide benefit to selected Cpc‐PH patients.^14^, ^15^ Conversely, of the vasodilators prescribed for non‐SCC Ipc‐PH patients, most were started inappropriately, potentially contributing to increased hospitalizations.

This study did not demonstrate a mortality benefit related to SCC care for PH‐HFpEF patients as a whole or their hemodynamic subgroups. This may not be surprising, given the dearth of guideline‐directed medications in PH‐HFpEF and the challenges to manage many of the contributing comorbidities.

This study had limitations. While our SCC was a single‐center, we compared to a variety of non‐SCC locations within the UPMC system. Multicenter SCC trials will be needed to fully define benefits nationally or globally. Medication usage was only collected on yearly intervals which could affect the medication assessment. Finally, future work to subclassify these patients into more specific cohorts of PH‐HFpEF could aid in identifying those who benefit the most from SCC care and potentially new therapies, including SGLT2 inhibitors.^16^

In conclusion, these findings offer quantitative proof to bolster efforts to refer PH‐HFpEF patients to SCC with demonstrated improvement in hospitalizations, likely related to medication use and increased guideline‐directed disease monitoring.

## CONFLICT OF INTERESTS

S. Y. C. has served as a consultant for United Therapeutics and Acceleron Pharma; S. Y. C. is a director, officer, and shareholder in Synhale Therapeutics; S. Y. C. has held research grants from Actelion, Bayer, and Pfizer. S. Y. C. has filed patent applications regarding the targeting of metabolism in pulmonary hypertension. The remaining authors declare that there are no conflict of interests.

## ETHICS STATEMENT

The Institutional Review Board at the University of Pittsburgh Medical Center (UPMC) approved this study (PRO11070366), and this study was performed in accordance with the Helsinki Declaration.

## AUTHOR CONTRIBUTIONS

Stephen Y. Chan is the guarantor of this project, had full access to the data in the study, and takes responsibility of the content of the manuscript including the integrity of the data and the accuracy of the data analysis. Chad M. Kosanovich, Hongyang Pi, Mehdi Nouraie, and Stephen Y. Chan conceived and designed the study. Adam Handen, Erin Schikowski, and Yimin Chen performed clinical data collection from electronic health records. Floyd W. Thoma, Steve Koscumb, and Suresh Mulukutla obtained medication collection from the electronic health records. Mehdi Nouraie performed statistical analyses. Chad M. Kosanovich, Hongyang Pi, Mehdi Nouraie, and Stephen Y. Chan drafted the manuscript. All authors provided input on final letter revision.
